# On the Nature of Clitics and Their Sensitivity to Number Attraction Effects

**DOI:** 10.3389/fpsyg.2017.01470

**Published:** 2017-09-05

**Authors:** Mikel Santesteban, Adam Zawiszewski, Kepa Erdocia, Itziar Laka

**Affiliations:** Department of Linguistics and Basque Studies, University of the Basque Country (UPV/EHU) Vitoria-Gasteiz, Spain

**Keywords:** clitics, agreement, pronouns, object agreement, attraction effects, sentence processing, cue-based retrieval

## Abstract

Pronominal dependencies have been shown to be more resilient to attraction effects than subject-verb agreement. We use this phenomenon to investigate whether antecedent-clitic dependencies in Spanish are computed like agreement or like pronominal dependencies. In Experiment 1, an acceptability judgment self-paced reading task was used. Accuracy data yielded reliable attraction effects in both grammatical and ungrammatical sentences, only in singular (but not plural) clitics. Reading times did not show reliable attraction effects. In Experiment 2, we measured electrophysiological responses to violations, which elicited a biphasic frontal negativity-P600 pattern. Number attraction modulated the frontal negativity but not the amplitude of the P600 component. This differs from ERP findings on subject-verb agreement, since when the baseline matching condition obtained a biphasic pattern, attraction effects only modulated the P600, not the preceding negativity. We argue that these findings support cue-retrieval accounts of dependency resolution and further suggest that the sensitivity to attraction effects shown by clitics resembles more the computation of pronominal dependencies than that of agreement.

## Introduction

Discovering the dependency relations between different elements of a sentence allows us to disentangle its meaning. In these dependency relations, verbal or nominal constituents match in certain features (i.e., number, person and/or gender) with another constituent of the sentence (Corbett, 2006). One of the most frequently studied dependency is that between a subject and a verb, where the features of the subject (e.g., the number) determine the form of the verb (e.g., *the key is…* vs. *the keys are…*) (see Bock and Middleton, 2011 for a review). In this paper we investigate a type of syntactic dependency that has received little attention in psycholinguistics: antecedent-clitic relations. There is debate in linguistics regarding the nature of clitics, where clitics are argued to be either pronouns or agreement morphemes. Our main objective is to experimentally explore the nature of antecedent-clitic dependencies. For that purpose, we use *agreement attraction*, a phenomenon showing that the presence of alternative candidates can disrupt the computation of dependency relations between two elements (Bock and Miller, 1991; Nicol et al., 1997). More specifically, we explore whether antecedent-clitic dependencies show similar behavioral (Experiment 1) and electrophysiological (Experiment 2) patterns of number agreement attraction as those previously reported for subject-verb agreement relations or as those reported for antecedent-pronoun relations.

### Why Antecedent-Clitic Dependencies?

The nature of Romance clitics has been much debated in Generative Linguistics since the seminal works by Kayne (1975) and Zwicky (1977), but experimental evidence regarding how they are processed is scarce. The status of clitics and particularly Romance clitics are an important subject of research in generative linguistics due to their intermediate/mixed behavior between independent pronouns and affixed agreement morphemes. In the case of the Spanish object-clitics we studied, they agree with their antecedent in number [*Anna vió la novela_*fem.sg*_/las novelas_*fem.pl*_ y la_fem.sg/_las_*fem.pl*_ compró;* “Anna saw the novel_*fem.sg*_/s_*fem.pl*_ and (she) bought it_*fem.sg*_/them_*fem.pl*_”], and gender [*Anna vió el libro_masc.sg_/los libros_masc.pl_ y lo_masc.sg/_los_masc.pl_ compró;* “Anna saw the book/s and (she) bought it_*masc.sg*_/them_*masc.pl*_”], unlike verbal inflection that agrees in person and number. These object-clitics correspond to the object arguments of the sentences’ main verb *comprar* (“to buy”). Hence, like pronouns, Spanish object clitics agree in gender and not person, satisfy verbal subcategorization properties and behave as arguments of the verb. However, like agreement (inflectional) morphemes, clitics are unstressed and affixed to the verb. In generative linguistics, there are two main competing approaches accounting for the nature of clitics:

Kayne (1975) originally proposed that clitics were syntactically independent elements in what we will refer to as the *Clitics as Pronouns Hypothesis*: clitics are pronoun noun phrases (NPs) generated at argument position that attach to the verb in the course of the derivation. In this view, NP-clitic dependencies are a case of referential co-dependency and the clitic occupies the argument position (Torrego, 1988; Uriagereka, 1995; Sportiche, 1998; Anagnostopoulou, 2003; Marchis and Alexiadou, 2013, among others). In a variant of this hypothesis, the clitic is generated in its surface position, while the argument position is filled by the empty pronominal *pro* (Strozer, 1976; Rivas, 1977; Jaeggli, 1982; Borer, 1984; among others). On the other hand, according to what we will refer to as the *Clitics as Agreement Hypothesis*, pronominal clitics are agreement morphemes, part of Inflection and not generated in argument position (e.g., Jaeggli, 1986; Suñer, 1988; Fernández Soriano, 1989; Monachesi, 2005 among others).

Our main objective is to contribute to better understanding the nature of clitics by testing whether and to what extent the behavioral and electrophysiological pattern found during clitic processing resembles that reported previously in the literature for verb agreement, or whether it aligns better with the processing patterns of pronominal concord. To that end, we explore (i) whether, in behavioral measures, antecedent-clitic dependencies are prone to number attraction effects similar to those found in subject-verb agreement, or whether they are more resilient to these effects as antecedent-pronoun dependencies are (see further discussion about this issue in next section); and (ii) whether, in electrophysiological measures, they elicit the same electrophysiological indexes of attraction as those previously reported for subject-verb agreement.

### On Number Attraction Effects

The study of the contexts where attraction phenomena occur during language production has shed light on the main factors involved in agreement processing: in sentence preambles such as *The key to the cabinet(s)…*, speakers produce more number agreement errors completing preambles containing an *attractor* noun that does not match (i.e., *cabinets*) in number with the agreement controller (i.e., the head noun *key*), than when the attractor matches (Bock and Miller, 1991; see Bock and Middleton, 2011; Franck, 2011 for exhaustive reviews of attraction effects in various types of agreement dependencies). Research on attraction effects in language comprehension is much more scarce than in production, and it has considered almost exclusively subject-verb agreement (in English: Nicol et al., 1997; Pearlmutter et al., 1999; Pearlmutter, 2000; Wagers et al., 2009; Shen et al., 2013; in Dutch: Kaan, 2002; Chen et al., 2007; Severens et al., 2008; in Spanish: Acuña-Fariña et al., 2014; Lago et al., 2015; in French: Franck et al., 2015). However, recent studies have also explored antecedent-reflexive pronoun concord (Dillon et al., 2013, 2014, 2016; Jäger et al., 2015; Patil et al., 2016; Parker and Phillips, 2017; for a thorough literature review on attraction effects in subject-verb and antecedent-pronoun dependencies, see Jäger et al., 2017).

Early studies adopted the *feature percolation hypothesis* postulated to account for attraction effects in language production. According to this account, attraction effects in both production and comprehension occur because the number features of the attractor noun can erroneously percolate over the number features of the agreement controller, which results in an erroneous number representation of the agreement controller (e.g., Nicol et al., 1997; Pearlmutter et al., 1999; Pearlmutter, 2000).

More recently, it has been proposed that attraction effects are best accounted for by means of a similarity-based interference model (Badecker and Kuminiak, 2007; Wagers et al., 2009; see Dillon et al., 2013; Jäger et al., 2017; for computational simulations of the model) inspired in the ACT-R model (Lewis and Vasishth, 2005). According to this model, dependency relations are established by retrieving from memory the agreement dependents. When the agreeing element (e.g., a verb or a clitic) is encoded, it engages a cue-based retrieval mechanism to search for a matching controller in memory. But this retrieval mechanism is susceptible to similarity-based interference from other items in memory. Hence, when a distracting element that carries similar features (e.g., semantic, structural features) as the controller is present in the sentence, interference occurs because the distracting element might be misidentified as the controller. Importantly, this model predicts attraction effects to be only present or to be larger during the processing of ungrammatical than grammatical sentences. Wagers et al. (2009) suggested two options for cue-retrieval mechanisms to account for these asymmetric effects: (a) encountering the agreeing element engages retrieval mechanisms that retrieve number-matching NPs but (almost) never retrieve partially matching ones (i.e., a number mismatching attractor in grammatical sentences); or (b) the correct agreeing element form is predicted after encountering the controller NP and the cue-based reanalysis process ensues almost exclusively when ungrammaticality is detected.

However, several studies report the presence of number attraction effects in both grammatical and ungrammatical sentences. In these studies, sentence acceptability, self-paced reading for comprehension and eye-tracking measures showed that participants are slower reading or accepting grammatical sentences with a singular subject and a plural attractor (e.g., *The author of the speeches was…* vs. *The author of the speech was…*) than accepting sentences where both NPs were singular (Nicol et al., 1997; Pearlmutter et al., 1999; Pearlmutter, 2000; Acuña-Fariña et al., 2014). In contrast, for ungrammatical sentences, mismatching attractors have been shown to elicit faster reading times as compared to matching ones in self-paced reading tasks (Pearlmutter et al., 1999; Wagers et al., 2009; Franck et al., 2015; Lago et al., 2015) and eye-tracking measures (Dillon et al., 2013). That is, attraction effects interfere in the processing of the agreement controller in grammatical sentences but facilitate it in ungrammatical ones. However, Wagers et al. (2009) identified a confound variable that might have led to the interference attraction effects reported in grammatical sentences: since in all these studies attractors and agreeing verbs where adjacent, the interference effects observed at the verb might be due to carry-over effects of the slower times needed to process the morphologically marked plural rather than unmarked singular attractors.

Nevertheless, in a recent study, Franck et al. (2015) showed both facilitation and interference attraction effects in grammatical sentences were the attractor and the verb were not adjacent and they suggested that experimental design factors might affect the direction of the effect. In a self-paced reading for comprehension task in French only including grammatical sentences (Experiment 1), they reported attraction facilitation effects. In contrast, in a speeded acceptability judgment task, participants showed attraction interference effects (slower acceptability judgments) when judging both grammatical and ungrammatical sentences containing number mismatching attractors, as compared to matching ones. Franck et al. (2015) interpreted their results as evidence that different behavioral tasks tap into different processes: while self-paced reading taps structure building processes, grammaticality judgment taps into later processes of agreement computation. Either way, the fact that attraction effects were detected in both grammatical and ungrammatical sentences might support feature percolation accounts.

However, many recent studies found reliable attraction effects in ungrammatical sentences but not in grammatical ones, favoring similarity-based interference accounts (Wagers et al., 2009; Dillon et al., 2013; Lago et al., 2015). This *grammatical* vs. *ungrammatical asymmetry* of attraction effects was interpreted as the main evidence that attraction effects are mainly due to similarity-based interference effects during the retrieval of the cues necessary to build dependency relations (e.g., Lewis and Vasishth, 2005), and not due to a faulty representation of the agreement controller, as suggested by the feature percolation account. As reviewed in the next section, electrophysiological evidence of attraction effects replicated the grammatical asymmetry of attraction effects (e.g., Kaan, 2002; Shen et al., 2013; Tanner et al., 2014, 2016).

Morphological markedness plays a crucial role during agreement attraction in comprehension: attraction effects are either only found in singular, but not plural agreement (Nicol et al., 1997; Wagers et al., 2009, in acceptability and self-paced reading data), or are larger in singular than plural agreement (Acuña-Fariña et al., 2014, in eye-tracking measures). These findings replicate the number markedness effects also reported in production studies (Bock and Miller, 1991; Bock and Cutting, 1992; Bock and Eberhard, 1993; Eberhard, 1997), suggesting that morphologically marked plural distractors are stronger attractors than non-marked singular ones in both modalities. Thus, attraction effects might sometimes be obscured and delayed due to carry-over effects of plural attractors when the attractor and the agreeing element are adjacent. However, those carry-over effects do not last long: they can be avoided by including a word between the attractor and the verb (e.g., Wagers et al., 2009) and even when the attractor and the verb are adjacent, attraction effects are detected at the region following the verb (Pearlmutter et al., 1999).

All research reviewed above studied subject-verb agreement dependencies. But do attraction effects also affect the processing, and more particularly the comprehension of antecedent-pronoun dependencies? In production, pronoun-antecedent agreement seems to be as sensitive to attraction effects as subject-verb agreement is, but the former is more sensitive to notional number factors (e.g., Bock et al., 1999, 2004), suggesting that pronominal dependencies may rely more on the retrieval of the semantic/lexical representation of the antecedent. In comprehension, early studies exploring the role of grammatical constraints in antecedent-reflexive pronoun gender agreement showed that they are resilient to interference from other possible antecedent candidates (Nicol and Swinney, 1989; Sturt, 2003; *inter alia*). More recently, these findings have been replicated in studies that compared the magnitude of attraction effects in antecedent-reflexive pronoun vs. subject-verb agreement dependencies. In a reading for comprehension eye-tracking experiment, Dillon et al. (2013) showed reliable attraction effects for subject-verb agreement (shorter total reading times and fewer regressions to the critical agreement region were obtained in sentences containing mismatching attractors as compared to sentences containing matching ones, but only in ungrammatical sentences, replicating the grammatical asymmetry of attraction). No signs of attraction effects were found for reflexive pronouns (e.g., *The new executive who oversaw the middle manager/s apparently doubted himself/^∗^themselves…*). Dillon et al. (2013) interpreted the resilience of reflexive pronouns to attraction effects as evidence that subject-verb vs. antecedent-reflexive pronoun dependencies involve qualitatively different processes (see also Phillips et al., 2011). According to the authors, these different linguistic dependencies are sensitive to different linguistic features: (a) verbal agreement is a formal morphosyntactic mechanism to index the arguments of the verb, and feature retrieval is mainly driven by ranked morphological and structural cues (i.e., number feature and subjecthood cues, respectively); and (b) pronominal concord is a dependency between two NPs and therefore antecedent retrieval is driven by syntactic (structural) cues.

Interestingly, recent eye-tracking studies show that although (English) reflexives are more resilient to attraction, they are indeed susceptible to it. For instance, Patil et al. (2016) showed that when the role of structural-cues such as subjecthood is controlled (e.g., both the antecedent of the reflexive and the attractor were subjects), attraction effects occurred when the attractor mismatched in morphological cues such as gender. Parker and Phillips (2017) also showed that no attraction effects occurred when the attractor mismatched in a single feature (i.e., gender) with the antecedent, but they did when the attractor mismatched in two features (e.g., gender and animacy, number and animacy or number and gender). These authors suggested that both subject-verb and antecedent-reflexive pronoun agreement engage similar cue-based retrieval mechanisms. However, following Dillon et al. (2013), Parker and Phillips (2017) suggested that reflexive pronoun dependencies weight structural cues more strongly than morphological cues, which precludes the erroneous retrieval of non-licensed antecedent candidates (see also Dillon et al., 2014, 2016).

In sum, behavioral measures show that subject-verb agreement comprehension is prone to attraction effects (Nicol et al., 1997; Pearlmutter et al., 1999; Pearlmutter, 2000; Wagers et al., 2009; Acuña-Fariña et al., 2014; Franck et al., 2015; Lago et al., 2015), but antecedent-pronoun dependencies are more resilient to these effects (Dillon et al., 2013; Patil et al., 2016; Parker and Phillips, 2017; see also Jäger et al., 2017 for a thorough review and discussion). Although attraction effects have been also reported in grammatical sentences, they are stronger and more consistent in ungrammatical sentences (e.g., Pearlmutter et al., 1999; Wagers et al., 2009; Lago et al., 2015), supporting similarity-based accounts. Next, we review the main findings of ERP studies on agreement and number attraction effects.

### ERP Correlates of Syntactic Dependency Processing

In general, when processing syntactic violations in subject-verb, object-verb, or antecedent-pronoun dependencies, three types of electrophysiological correlates have been reported in the ERP literature: Left Anterior Negativity (LAN), N400 and a centro-parietal positivity (P600) (for a detailed description and interpretation of each component see i.e., Bornkessel and Schlesewsky, 2006).

Most studies observed biphasic patterns with negative components (LAN/N400) followed by a positive component (P600). Some studies reported a biphasic LAN – P600 pattern for subject-verb agreement violations (Kutas and Hillyard, 1983; Osterhout and Mobley, 1995; De Vincenzi et al., 2003; Silva-Pereyra and Carreiras, 2007, among others) as well as for determiner-noun or noun-adjective gender agreement violations (Gunter et al., 2000; Deutsch and Bentin, 2001; Barber and Carreiras, 2005; Martin-Loeches et al., 2006; Molinaro et al., 2008). Other studies reported a biphasic N400-P600 pattern for subject-verb and object-verb agreement violations (Coulson et al., 1998; Zawiszewski and Friederici, 2009; Díaz et al., 2011; Zawiszewski et al., 2011) as well as for antecedent-pronoun violations (Schmitt et al., 2002; Hammer et al., 2005, 2008; Lamers et al., 2006). Finally, some studies have also reported an isolated P600 component for subject-verb agreement violations (Osterhout et al., 1996; Nevins et al., 2007; Frenck-Mestre et al., 2008), for determiner-noun or noun-adjective gender agreement relations (Osterhout and Mobley, 1995; Osterhout et al., 1997; Foucart and Frenck-Mestre, 2011, 2012) and for antecedent-pronoun violations (Lamers et al., 2006, 2008; Silva-Pereyra et al., 2012; Xu et al., 2013; Rossi et al., 2014). As far as we know, no study has shown an isolated early negativity (N400 or LAN).

### ERP Correlates of Attraction Effects

Regarding the electrophysiological responses underlying number attraction effects, the available evidence is rather scarce and focused on subject-verb number agreement. To our knowledge, no study explored attraction effects in antecedent-clitic dependencies.

Electrophysiological indexes of attraction effects in subject-verb agreement are heterogeneous, but two main results have been observed: (a) electrophysiological indexes of agreement violation detection are less salient and harder to detect in sentences containing number mismatching attractors than matching attractors (Kaan, 2002; Chen et al., 2007; Severens et al., 2008; Shen et al., 2013; Tanner et al., 2014, 2016); and (b) the four studies that checked for asymmetrical attraction effects found an asymmetry: number mismatching attractors elicit a reduction of ERP components as compared to number matching ones in ungrammatical sentences, but not in grammatical ones (Kaan, 2002; Shen et al., 2013; Tanner et al., 2014, 2016).

Focusing on the studies that reported asymmetrical attraction effects, and thus support the cue-based retrieval account of agreement computation (Lewis and Vasishth, 2005; Wagers et al., 2009), Kaan (2002) investigated the effects of distance and number interference in subject agreement processing: Dutch participants performed an acceptability rating task in sentences containing subject and object NPs that either matched or mismatched in number. ERP responses following the critical verb revealed main grammaticality effects reflected by a bilateral negativity over central and posterior sites between 300 and 500 ms, and a P600 effect between 500–700 and 700–900 ms. A main number attraction effect was revealed by a significantly larger P600 component between 500 and 700 ms following subject agreement violations in sentences with only singular NPs (i.e., the control singular number matching condition) than in any other condition. Number mismatching attractors elicited a smaller P600 in singular subject agreement, but not in plural, replicating the number markedness effects (Eberhard, 1997; Nicol et al., 1997; Wagers et al., 2009). Finally, the modulation of the P600 related to attraction effects was asymmetrical, as it only occurred in ungrammatical sentences.

Tanner et al. (2014) provide behavioral and ERP evidence supporting the asymmetric pattern of attraction effects: English speaking participants showed a main P600 component elicited by subject-verb agreement violations, with attraction effects revealing a smaller P600 in sentences containing number mismatching attractors than number matching ones. These attraction effects were asymmetrical: Participants showed a reliable P600 effect and were less accurate judging ungrammatical sentences that contained number mismatching rather than number matching attractor NPs. In contrast, they showed no P600 effect and were similarly accurate while judging the acceptability of grammatical sentences containing number matching and mismatching attractor NPs. These results obtained both with and without an adverb intervening between the attractor noun and the auxiliary verb (*The chemist with the test tube(s) (probably) is/^∗^are…*), suggesting that ERP indexes of attraction are resilient to carry-over effects of the plural attractor. In a recent study, Tanner et al. (2016) replicated this pattern of attraction effects revealing that number mismatching attractors reduce the magnitude of the P600 as compared to number matching ones in ungrammatical sentences.

Shen et al. (2013) used a comprehension task where participants listened to several narrations in English with a low proportion of violations. In sentences with no attractor NPs, singular subject agreement violations elicited a bilateral frontal negativity between 150 and 300 ms (interpreted as a LAN) followed by a P600 between 700 and 950 ms. In sentences with complex NPs (e.g., *A catalog with color picture/s sit/^∗^sits…*), those containing number matching attractors elicited an atypical early posterior negativity between 150 and 300 ms, and no P600, while those containing number mismatching attractors elicited neither early posterior negativity nor P600 effects. Although the authors suggest that the posterior negativity resembles the timing and distribution of the N400, the distribution of the negativity related to morphosyntactic violations is rather frontal (and lateralized: LAN) and starts later on (300 ms after the stimulus onset) (see Molinaro et al., 2011, 2015; Tanner, 2015 for an extensive review and discussion). These different ERP patterns might be due to the naturalistic procedure used in this study (i.e., sentences were auditorily presented and embedded in discourse), as compared to the procedure used in most other studies. Regardless of the origin of the atypical early components in this study, the relevant fact for our discussion is that agreement attraction effects reached significance only in ungrammatical sentences (differences between sentences with number matching vs. mismatching attractor NPs), replicating the asymmetric pattern of attraction effects. Shen et al. (2013) interpreted these results as evidence that subject agreement is affected by the presence of number-bearing elements other than the subject itself, with number mismatching elements completely “masking” subject agreement violations.

There are two more studies that explored number attraction effects in subject-verb agreement, but they did not analyze whether these were asymmetric. In an acceptability rating task, Severens et al. (2008) explored in Dutch whether the ambiguity of the determiner of the controller NP affects number attraction. In number match conditions, an atypical ERP pattern related to morphological agreement violations was found, as subject agreement violations only elicited an N400, not followed by a P600. This was interpreted to reflect a blatant violation of the expected verb form during a first, syntactically shallow process that cannot be repaired by further analysis, resulting in the absence of a P600. In violations involving number mismatching conditions, only a P600 was elicited, which was interpreted as reflecting a deeper syntactic processing triggered by the strong conflict between a shallow syntactic analysis that suggests the first noun (singular) to be the controller and a combinatorial analysis that suggest the noun (plural) agreeing in number with the verb (i.e., the attractor) to be the controller. In other words, the agreement attraction effects were argued to prevent the generation of a N400 component correlated to the ungrammatical verb. Similar findings were reported by Chen et al. (2007) for English singular subject agreement, although this study reported a LAN instead of a N400. Here, a biphasic LAN-P600 pattern was observed in matching conditions, while only a P600 (but no LAN) was reported in mismatching conditions (i.e., *The price of the cars ^∗^were*…”).

In summary, the electrophysiological indexes of attraction are mainly reflected by a reduction of main ERP components related to agreement violation detection. The most consistent finding is the reduction of the later P600 component, found in three out of six studies (Kaan, 2002; Tanner et al., 2014, 2016). The other studies showed a reduction of diverse early components: a posterior early negativity (Shen et al., 2013), an N400 (Severens et al., 2008), or a LAN (Chen et al., 2007), but two showed atypical ERP components in the baseline number matching conditions, which might pose problems to the generalizability of attraction effect to other types of dependencies. Further research needs to bring some light on the origin of such heterogeneous patterns of attraction effects in subject-verb agreement. However, it is worth noting that all the studies that explored it found an asymmetrical pattern of attraction effects (Kaan, 2002; Shen et al., 2013; Tanner et al., 2014, 2016), which supports the similarity-based interference account of attraction (Lewis and Vasishth, 2005; Wagers et al., 2009).

## The Present Study

In the present study, we explore for the first time the behavioral and neurophysiological processes of number attraction when processing antecedent-object clitic dependencies in Spanish. We investigate whether antecedent-clitic dependencies are resilient to attraction effects with the aim to provide some experimental evidence on whether clitic dependencies are processed like an agreement dependency or a pronominal dependency.

We carried out two acceptability judgment experiments in Spanish. In each experiment, Spanish native speakers were presented with sentences that had an inanimate object NP containing a PP ([_NP_ Det N [_PP_ P [_NP_ Det N]]]). The Noun inside the PP either matched or mismatched in number with the Noun of the main NP. This complex NP was followed by a left-dislocated object clitic that either matched (grammatical) or mismatched (ungrammatical) in number with the antecedent NP. Clitic left-dislocated structures were investigated for the reason that in peninsular Spanish this is the only way to have the antecedent of the clitic in the same main sentence as the clitic. In this case, all our sentences contained an omitted subject that in its overt form would be placed between the object NP and the clitic (see **Table 1**)^1^. In Experiment 1, a self-paced reading task was used and singular and plural antecedent NPs were presented. In Experiment 2, singular antecedent NPs were presented and the acceptability ratings and electrophysiological responses of participants were recorded while reading sentences presented with a RSVP paradigm.

**Table 1:** Sample set of experimental items for Experiments 1 and 2.

	Conditions	
	
Sentences	Attractor number	Grammaticality
**Singular objects (Experiments 1 and 2)**		
(1) El cartero afirmó que el paquete para el vecino lo entregó a tiempo.	Singular (match)	Grammatical
(2) El cartero afirmó que el paquete para el vecino ^∗^los entregó a tiempo.	Singular (match)	Ungrammatical
“The postman stated that the package for the neighbor (he) delivered it/^∗^them on time”		
(3) El cartero afirmó que el paquete para los vecinos lo entregó a tiempo.	Plural (mismatch)	Grammatical
(4) El cartero afirmó que el paquete para los vecinos ^∗^los entregó a tiempo.	Plural (mismatch)	Ungrammatical
“The postman stated that the package for the neighbors (he) delivered it/^∗^them on time”		
**Plural objects (Experiment 1)**		
(5) El cartero afirmó que los paquetes para los vecinos los entregó a tiempo.	Plural (match)	Grammatical
(6) El cartero afirmó que los paquetes para los vecinos ^∗^lo entregó a tiempo.	Plural (match)	Ungrammatical
“The postman stated that the packages for the neighbors (he) delivered ^∗^it/them on time”		
(7) El cartero afirmó que los paquetes para el vecino los entregó a tiempo.	Singular (mismatch)	Grammatical
(8) El cartero afirmó que los paquetes para el vecino ^∗^lo entregó a tiempo.	Singular (mismatch)	Ungrammatical
“The postman stated that the packages for the neighbor (he) delivered ^∗^it/them on time”		


## Experiment 1

In Experiment 1 we explored whether the number attraction effects previously observed for subject-verb agreement (e.g., Nicol et al., 1997; Pearlmutter et al., 1999; Pearlmutter, 2000; Wagers et al., 2009) obtain during the processing of the dependency between antecedents and clitics. If this dependency is a subtype of agreement as suggested by the *Clitics as Agreement Hypothesis* we expect faster reading times and lower accuracy judging ungrammatical sentences containing antecedent NPs containing a number matching attractor NP (Wagers et al., 2009). Since, for the sake of completeness, we included singular and plural antecedents, we also expect to replicate the number markedness effects of attraction (Nicol et al., 1997; Wagers et al., 2009), so that larger attraction effects (if any) are expected for sentences containing singular antecedent NPs, than for sentences containing plural antecedent NPs. However, if clitics establish pronominal dependencies as argued by the *Clitics as Pronouns Hypothesis*, no attraction effects are expected in self-paced readings (Experiment 1), as suggested by previous evidence with other pronominal forms like reflexives (Dillon et al., 2013; Parker and Phillips, 2017). In sum, the presence of attraction effects suggests that antecedent-clitic dependencies are processed as a subtype of agreement.

At this point, we would like to add a cautionary note about the time course at which these effects are to be observed. In most self-paced reading studies, attraction effects appear in the region following the critical verb (Wagers et al., 2009; Lago et al., 2015). In our experimental sentences, as in Pearlmutter et al. (1999), the attractor NP immediately precedes the clitic, so that some attractor number carry-over effects are expected (Wagers et al., 2009). Hence, we expect attraction (and acceptability) effects to arise at the position following the critical word (CW), the clitic.

### Method

#### Participants

Sixty native speakers of Spanish (42 females, mean age years 22.7; *SD* = 5.8), undergraduates at the University of the Basque Country (UPV/EHU) were paid for their participation in the study. All Participants gave written informed consent under experimental protocols approved by the Ethics Committee of the UPV/EHU (Comité de Ética para las Investigaciones relacionadas con Seres Humanos, CEISH), in accordance with the Declaration of Helsinki.

#### Materials

Experimental materials consisted of 48 sentences. Each sentence had the following structure: a subject NP followed by the main verb and a subordinate clause containing an object NP + object-clitic + subordinate verb + PP (see **Table 1** and Appendix). Crucially, object NPs were third person and contained a singular or plural head noun and a singular or plural NP inside the modifying PP. Eight experimental conditions were created crossing three factors: Object Number (singular vs. plural) vs. Attractor Number (singular vs. plural) and Grammaticality (grammatical vs. ungrammatical sentences). Each sentence was presented once in each of these conditions.

Additionally, we created 96 filler sentences to introduce some variability in the stimuli. 84 of these filler sentences were grammatical and 12 contained subject-verb agreement violations. We created eight lists containing 144 sentences, from which 48 were experimental sentences (6 per condition) and 96 were fillers. Each list contained a total of 36 (25%) ungrammatical sentences. Each participant was presented with only one of these lists. Each item was presented only once in each list. Four additional sentences (2 grammatical and 2 ungrammatical) were used as practice trials.

#### Procedure

Linger (Rohde, 2001) software was used to present the stimuli. Before the experiment started, participants received written instructions about the main procedure. They were asked to read and understand sentences word-by-word as fast as they could by pressing the spacebar in a self-paced reading task. The materials were pseudo-randomized in the following way: no sentences of the same condition were displayed one after another and each experimental sentence (see examples 1–8 in **Table 1**) was followed by a filler sentence. A fixation cross (+) indicated the beginning of each trial. After each sentence a question mark was presented and participants were instructed to press one of two buttons (1 and 2 on the keyboard) depending on whether the previously displayed sentence was grammatical or not. Half of the participants pressed 1 for grammatical sentences and 2 for ungrammatical sentences; the other half used the reversed configuration. All 144 sentences were distributed over 4 blocks, and participants were asked to have short breaks between these blocks. Before the experiment began, participants were familiarized with the procedure by means of a short trial session in which 4 sentences were presented (2 grammatical, 2 ungrammatical sentences). The experiment lasted about 25 min.

#### Data Analysis

Acceptability judgment accuracy and reading time data were analyzed with mixed logit and linear mixed effects regression models, respectively. Reading times faster than 50 ms or slower than 4000 ms were excluded (0.5% of the data), and reading times that exceeded a threshold of 2.5 standard deviations by region and condition were excluded (2.3% of the analyzed data). Next, raw reading times were log-transformed to normalize the data and spill-over effects of the previous two words were calculated for each word region^2^.

In the analyses of grammaticality judgment and self-paced reading time data, our binomial variable (whether a grammaticality judgment was performed or not in the grammaticality judgment task), or log-transformed reading time dependent variables were fitted with (generalized) linear mixed regression models including crossed random and fixed effects (Baayen, 2008; Baayen et al., 2008; Jaeger, 2008). The following sum coded fixed factors were included in the models: Object Number (singular vs. plural), Attractor Number (singular vs. plural), Grammaticality (grammatical vs. ungrammatical), and their interactions. In the reading time analyses, the spill-over effects of the two previous words were also included in the model as fixed effects. When the maximal model failed to converge or showed high correlation parameters between random effects (>0.8), we used the backward selection based on χ^2^. Finally, whenever a significant interaction effect revealed different patterns of results for the involved fixed factors, we run simpler models that split without one of the involved fixed factors in order to find the source of the interaction (e.g., when the three-way interaction was significant, we run two separate models including the Attractor Number, Grammaticality, and their interactions as fixed factors; the maximal random effect structures of the main models was kept). All analyses were carried out in R (version 3.4.0; R Development Core Team, 2013) using the lmerTest package.

### Results

#### Grammaticality Judgment Errors (See **Table 2**)

**Table 2:** Raw count of errors (from a total of 360 responses per condition; percentages in brackets) and reaction time (ms) values of participants’ performance in the grammaticality judgment task in each experimental condition of Experiment 1.

	Grammaticality judgment errors		Response latencies	
		
	Grammatical	Ungrammatical	Grammatical	Ungrammatical
**Singular object**				
Singular attractor (match)	22 (6.1%)	15 (4.2%)	591	544
Plural attractor (mismatch)	67 (18.6%)	57 (15.8%)	603	570
*Attraction effect*	*-45 (-12.5%)*	*-42 (-11.6%)*	*-11*	*-26*
**Plural object**				
Plural attractor (match)	38 (10.6%)	34 (9.4%)	583	556
Singular attractor (mismatch)	51 (14.2%)	23 (6.4%)	586	589
*Attraction effect*	*-13 (-3.6%)*	*11 (3.0%)*	*-3*	*-33*


The maximal random effect structure justified by model comparison included a by-participant Grammaticality random slope. The results showed significant Attractor Number (β = 0.370, *SE* = 0.070, *z* = 5.224, *p* < 0.001), and Grammaticality (β = -0.281, *SE* = 0.126, *z* = -2.215, *p* = 0.026) effects. These effects showed that more errors were produced in grammatical than ungrammatical sentences and in sentences where the number of the antecedent NP and the attractor mismatched than matched.

There was also a significant Attractor Number by Object Number interaction (β = -0.390, *SE* = 0.070, *z* = -5.506, *p* < 0.001), revealing larger attraction effects (more errors judging sentences with number mismatching than matching attractors) in sentences containing singular than plural objects. The simpler models revealed that the attraction effects were only significant in sentences containing a singular object (β = 0.766, *SE* = 0.106, *z* = 7.168, *p* < 0.001) but not in sentences containing a plural object (β = -0.022, *SE* = 0.096, *z* = -0.235, *p* < 0.814). Finally, the three-way interaction was marginally significant (β = -0.130, *SE* = 0.070, *z* = -1.846, *p* < 0.064), revealing different grammaticality by attraction patterns in sentences containing singular and plural objects. The simpler models revealed a non-significant Attractor Number by Grammaticality interaction in sentences containing singular objects (β = 0.080, *SE* = 0.106, *z* = 0.758, *p* < 0.448), but a significant interaction for sentences with plural objects (β = -0.218, *SE* = 0.096, *z* = -2.260, *p* < 0.023). The later interaction revealed different direction of attraction effects in grammatical and ungrammatical conditions, but these effects were not significant in either condition (all *p*s > 0.10). No further effects were found (all *z* < 2).

#### Grammaticality Judgment Response Latencies (See **Table 2**)

None of the effects were significant (all *t*s < 2).

#### Self-paced Reading Response Latencies

**FIGURE 1:**
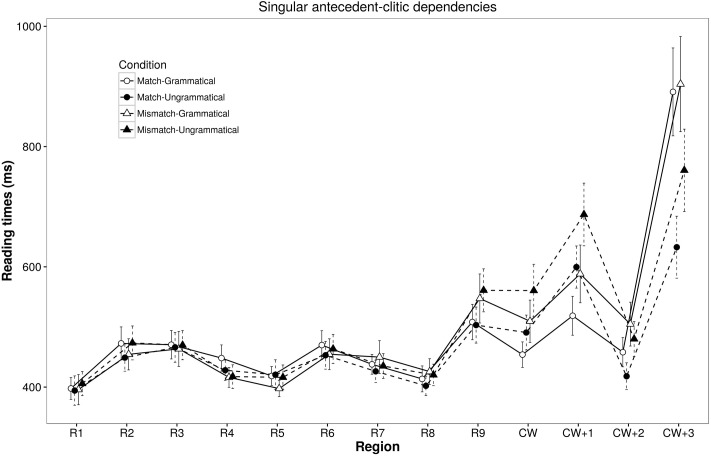
Self-paced reading results of sentences with *singular object nouns* (Experiment 1). Region by region means segregated by object noun number and grammaticality. The bars associated with each mean represent standard errors. Sample sentence: *El*_(R1)_ *cartero*_(R2)_ *afirmó*_(R3)_ *que*_(R4)_ *el*_(R5)_ *paquete*_(R6)_ *para*_(R7)_ *el/los*_(R8)_ *vecino(s)*_(R9)_ *lo/^∗^los*_(CW)_ *entregó*_(CW+1)_ *a*_(CW+2)_ *tiempo*_(CW+3)_.

**FIGURE 2:**
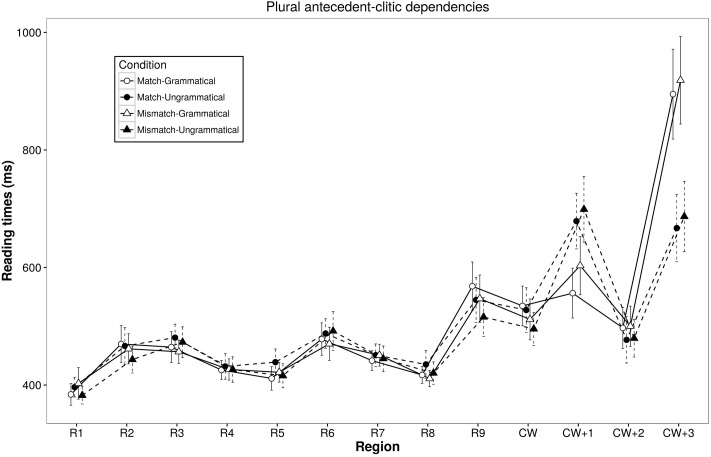
Self-paced reading results of sentences with *plural object nouns* (Experiment 1). Region by region means segregated by object noun number and grammaticality. The bars associated with each mean represent standard errors. Sample sentence: *El*_(R1)_ *cartero*_(R2)_ *afirmó*_(R3)_ *que*_(R4)_ *los*_(R5)_ *paquetes*_(R6)_ *para*_(R7)_ *el/los*_(R8)_ *vecino(s)*_(R9)_ *^∗^lo/los*_(CW)_ entregó_(CW+1)_ *a*_(CW+2)_ *tiempo*_(CW+3)_.

The maximal random effect structures justified by model comparison that did not have convergence or high correlation parameter problems did not include any random slopes for regions R5, R6, R7, CW+2, and CW+3 and contained a by-item Attractor Number random slope for regions R8, R9, CW, and CW+1^3^ (see **Tables 3**, **4**, for the self-paced reading data, reported in milliseconds, and the mixed-effect model based on log-transformed reading times, respectively). The main effect of Object Number was significant at the object region and marginally significant at the following region (R6 and R7) as well as at the two regions after the clitic (CW+1 and CW+2), with slower reading times in sentences containing plural than singular objects. The main effect of Grammaticality was marginally significant at the region after the object noun, which must be random, but most importantly it was fully significant at the region after the clitic (CW+1), revealing that participants were slower reading ungrammatical than grammatical sentences. This Grammaticality effect reversed in the last two regions of the sentence (CW+2 and CW+3). In this regard, the significant Grammaticality by Object Number interaction found at the clitic region revealed that this grammaticality effect was already present at the clitic region in sentences containing singular objects (β = 0.024, *SE* = 0.008, *t* = 2.773, *p* = 0.005), while it was not significant in sentences containing plural objects (*p* > 0.3) (see **Figures 1**, **2**). This interaction was also found in the region preceding the clitic region where violations might occur (R9), and it only signals the presence of non-significant random trends of opposite effects of grammaticality in sentences with singular vs. plural objects (both *p*s > 0.1). This effect must be random and is not further discussed.

**Table 3:** Self-paced reading results (in ms) in each experimental condition of Experiment 1.

	Rl	R2	R3	R4	R5	R6	R7	R8	R9	CW	CW+1	CW+2	CW+3
**Singular object**													
Match-grammatical	391	459	467	435	418	461	438	401	490	450	496	441	703
Match-ungrammatical	385	445	457	428	412	446	426	402	487	480	577	418	555
*Grammaticality effect*	*7*	*14*	*10*	*7*	*7*	*16*	*12*	*-1*	*4*	*-30*	*-80*	*23*	*148*
Mismatch-grammatical	382	443	444	416	398	442	433	422	495	485	517	472	715
Mismatch-ungrammatical	401	456	464	410	401	459	428	416	538	516	600	461	608
*Grammaticality effect*	*-19*	*-12*	*-20*	*6*	*-4*	*-17*	*4*	*6*	*-42*	*-31*	*-84*	*11*	*107*
*Attraction effect (Gramm)*	*9*	*15*	*23*	*19*	*21*	*20*	*5*	*-21*	*-5*	*-35*	*-20*	*-31*	*-12*
*Attraction effect (Ungramm)*	*-16*	*-11*	*-6*	*18*	*11*	*-13*	*-2*	*-14*	*-51*	*-36*	*-23*	*-43*	*-53*
**Plural object**													
Match-grammatical	379	448	454	426	405	467	442	417	524	507	521	472	680
Match-ungrammatical	396	449	482	424	429	480	447	423	511	494	620	433	557
*Grammaticality effect*	*-17*	*-1*	*-28*	*1*	*-24*	*-14*	*-6*	*-6*	*13*	*13*	*-99*	*39*	*123*
Mismatch-grammatical	387	447	457	421	422	456	446	411	509	478	512	472	712
Mismatch-ungrammatical	383	438	465	412	410	470	438	412	490	487	612	458	581
*Grammaticality effect*	*4*	*9*	*-8*	*9*	*12*	*-14*	*8*	*-1*	*19*	*-9*	*-99*	*14*	*132*
*Attraction effect (Gramm)*	*-8*	*0*	*-3*	*4*	*-17*	*11*	*-5*	*6*	*15*	*29*	*9*	*0*	*-32*
*Attraction effect (Ungramm)*	*14*	*10*	*17*	*12*	*19*	*11*	*9*	*12*	*21*	*7*	*9*	*-25*	*-24*


*R, region; CW, critical word (object-clitic). Region by region means segregated by object number and grammaticality and main grammaticality effects (grammatical minus ungrammatical conditions) and attraction effects (match minus mismatch conditions). Sample sentence (singular object conditions): El_*(R1)*_ cartero_*(R2)*_ afirmó_*(R3)*_ que_*(R4)*_ el_*(R5)*_ paquete_*(R6)*_ para_*(R7)*_ el/los_*(R8)*_ vecino(s)_*(R9)*_ lo/^∗^los_*(CW)*_ entregó_*(CW*+*1*)_ a_*(CW*+*2)*_ tiempo_*(CW*+*3)*_.*

**Table 4:** Linear mixed models for the analysis of the self-paced log-transformed reading times per region in Experiment 1.

Predictor	β	SE	*t*-value	*p*
**R5**				
(Intercept)	2.897	0.115	25.132	<0.001
Object number	0.007	0.005	1.454	0.146
Attractor number	-0.003	0.005	-0.593	0.553
Grammaticality	0.005	0.005	1.118	0.263
Attractor number × Object number	0.003	0.005	0.536	0.591
Grammaticality × Object number	0.002	0.005	0.323	0.746
Attractor number × Grammaticality	-0.006	0.005	-1.243	0.213
3-way interaction	-0.012	0.005	-2.439	0.014
Spillover_1	0.292	0.017	16.867	<0.001
Spillover_2	0.219	0.015	14.910	<0.001
**R6**				
(Intercept)	2.094	0.144	14.556	<0.001
Object number	0.013	0.006	2.347	0.019
Attractor number	-0.002	0.006	-0.394	0.694
Grammaticality	0.002	0.006	0.331	0.741
Attractor number × Object number	-0.007	0.006	-1.284	0.199
Grammaticality × Object Number	0.003	0.006	0.471	0.637
Attractor number × Grammaticality	0.008	0.006	1.442	0.149
3-way interaction	-0.002	0.006	-0.271	0.786
Spillover_1	0.387	0.021	18.541	<0.001
Spillover_2	0.276	0.021	13.300	<0.001
**R7**				
(Intercept)	3.094	0.112	27.639	<0.001
Object number	0.008	0.005	1.726	0.084
Attractor number	0.005	0.005	1.110	0.267
Grammaticality	-0.009	0.005	-1.896	0.058
Attractor number × Object Number	-0.005	0.005	-1.018	0.308
Grammaticality × Object Number	0.003	0.005	0.632	0.527
Attractor number × Grammaticality	-0.001	0.005	-0.274	0.784
3-way interaction	-0.003	0.005	-0.568	0.569
Spillover_1	0.213	0.016	13.747	<0.001
Spillover_2	0.275	0.019	14.755	<0.001
**R8**				
(Intercept)	3.123	0.106	29.527	<0.001
Object number	0.003	0.004	0.732	0.464
Attractor Number	0.004	0.005	0.898	0.373
Grammaticality	0.002	0.004	0.649	0.516
Attractor number × Object number	-0.010	0.004	-2.286	0.022
Grammaticality × Object number	0.002	0.004	0.556	0.578
Attractor number × Grammaticality	-<0.001	0.004	-0.149	0.881
3-way interaction	0.001	0.004	0.285	0.775
Spillover_1	0.254	0.016	15.323	<0.001
Spillover_2	0.217	0.014	15.502	<0.001
**R9**				
(Intercept)	1.913	0.165	11.538	<0.001
Object number	-0.002	0.006	-0.320	0.749
Attractor number	0.003	0.006	0.486	0.629
Grammaticality	-<0.001	0.006	-0.047	0.962
Attractor number × Object number	-0.019	0.006	-3.052	0.002
Grammaticality × Object number	-0.013	0.006	-2.149	0.031
Attractor number × Grammaticality	0.004	0.006	0.638	0.523
3-way interaction	-0.003	0.006	-0.599	0.549
Spillover_1	0.356	0.024	14.636	<0.001
Spillover_2	0.350	0.023	15.086	<0.001
**CW**				
(Intercept)	3.469	0.147	23.547	<0.001
Object number	0.008	0.006	1.318	0.187
Attractor number	0.008	0.006	1.214	0.230
Grammaticality	0.008	0.006	1.315	0.188
Attractor number × Object number	-0.023	0.006	-3.645	<0.001
Grammaticality × Object number	-0.016	0.006	-2.629	0.008
Attractor number × Grammaticality	0.001	0.006	0.260	0.794
3-way interaction	-<0.001	0.006	-0.039	0.968
Spillover_1	0.189	0.017	10.884	<0.001
Spillover_2	0.249	0.024	10.253	<0.001
**CW+1**				
(Intercept)	3.456	0.159	21.618	<0.001
Object number	0.014	0.007	1.895	0.058
Attractor number	0.020	0.008	2.563	0.013
Grammaticality	0.070	0.007	9.256	<0.001
Attractor number × Object number	-0.001	0.007	-0.236	0.813
Grammaticality × Object number	0.004	0.007	0.571	0.568
Attractor number × Grammaticality	-0.003	0.007	-0.450	0.652
3-way interaction	-0.006	0.007	-0.854	0.393
Spillover_1	0.188	0.021	8.562	<0.001
Spillover_2	0.269	0.020	13.159	<0.001
**CW+2**				
(Intercept)	5.283	0.142	37.285	<0.001
Object number	0.013	0.007	1.810	0.070
Attractor number	0.026	0.007	3.631	<0.001
Grammaticality	-0.035	0.007	-4.828	<0.001
Attractor number × Object number	-0.014	0.007	-1.930	0.053
Grammaticality × Object number	0.004	0.007	0.594	0.552
Attractor number × Grammaticality	0.011	0.007	1.506	0.132
3-way interaction	-0.006	0.007	-0.834	0.404
Spillover_1	0.015	0.016	0.926	0.354
Spillover_2	0.111	0.020	5.573	<0.001
**CW+3**				
(Intercept)	2.620	0.217	12.060	<0.001
Object number	-0.008	0.010	-0.820	0.412
Attractor number	0.011	0.010	1.103	0.270
Grammaticality	-0.136	0.010	-13.249	<0.001
Attractor number × Object number	-0.002	0.010	-0.178	0.858
Grammaticality × Object number	-0.005	0.010	-0.542	0.588
Attractor number × Grammaticality	0.009	0.010	0.876	0.380
3-way interaction	-0.017	0.010	-1.719	0.085
Spillover_1	0.375	0.027	13.918	<0.001
Spillover_2	0.250	0.023	10.720	<0.001


*R, region; CW, critical word (clitic). Sample sentence for the analyzed regions (singular object conditions): (…) el(_*R5*_) paquete(_*R6*_) para(_*R7*_) el/los(_*R8*_) vecino(s)(_*R9*_) lo/^∗^los(_*CW*_) entregó(_*CW*+*1*_) a(_*CW*+*2*_) tiempo(_*CW*+*3*_).*

The main effect of Attractor Number was significant at the post-clitic region and the following region (CW+1 and CW+2), showing that participants were slower reading sentences containing attractors that mismatched rather than matched in number with the object.

The Attractor Number by Object Number interaction was significant at the regions of the determiner of the attractor, the attractor and the clitic (R8, R9, CW), as well as marginally significant two regions after the clitic (CW+2). This interaction seems to reveal plural marking slow down effects (and carry-over of such effects) rather than number attraction effects. This is because, in the case of sentences containing singular objects, participants showed slower reading times in sentences containing number mismatching plural rather than number matching singular attractors (R8: β = 0.014, *SE* = 0.007, *t* = 2.096, *p* = 0.041; R9: β = 0.022, *SE* = 0.010, *t* = 2.196, *p* = 0.033; CW: β = 0.024, *SE* = 0.008, *t* = 2.773, *p* < 0.001; CW+2: β = 0.037, *SE* = 0.009, *t* = 3.941, *p* < 0.001). However, in sentences with plural objects, no attractor number effects were found in regions R8, CW and CW+2 (all *p*s > 0.10), and a marginally significant reversed attraction effect was only found at the region preceding the clitic (R9: β = -0.015, *SE* = 0.009, *t* = -1.725, *p* = 0.085), with faster reading times in sentences containing number mismatching singular rather than number matching plural attractors.

The Grammaticality by Attractor Number two-way interaction was not significant at any region. The three-way interaction was significant at R5, which must have been random, and was marginally significant at the last region at which wrap-up effects occur.

### Discussion

The grammaticality judgment accuracy data replicated two of the most common findings in agreement: (a) *An attraction effect*: participants produced more grammaticality judgment errors when sentences contained an attractor that mismatched the number of the antecedent NP as compared to sentences containing a number matching attractor (Nicol et al., 1997; Franck et al., 2015); (b) *A markedness effect*: attraction effects obtained with singular but not with plural antecedent NPs (Bock and Miller, 1991; Eberhard, 1997; Pearlmutter et al., 1999; Pearlmutter, 2000; Wagers et al., 2009). Plural attractors disrupted participants’ grammaticality judgment accuracy both when accepting grammatical sentences and when rejecting ungrammatical ones. This replicates the finding of attraction effects in the judgment of grammatical sentences by Nicol et al. (1997: Experiment 2), in contrasts with the results of Franck et al. (2015: Experiment 3).

Reading time results are less conclusive. This is because, despite the inclusion of spill-over effects in the model, the main number attraction effects seem to reflect carry-over effects of the larger difficulty of processing the number of the plural attractor presented just before the clitic (Pearlmutter et al., 1999; Wagers et al., 2009): in sentences containing singular antecedent NPs, the presence of plural attractors, as compared to singular ones, slowed down participants’ reading times. This effect persisted at the clitic region and the following ones, both in grammatical and ungrammatical sentences. In contrast, in sentences containing plural antecedent NPs, reading times at the attractor noun region were slower for matching plural than mismatching singular attractors. These slow down effects occurred only in grammatical sentences, and persisted only until the following clitic region. The fact that these effects appear in sentences with singular and plural antecedent NPs at the regions where the attractor is presented suggests that part, if not all, of the attractor number effects are due to the greater reading and processing cost of morphologically marked plural attractors. Consequently, we argue that these effects are not *bona fide* agreement attraction effects.

Importantly, similar grammaticality effects obtained while reading sentences containing singular or plural antecedent object NPs. In both cases, the presence of clitics mismatching in number with their antecedent NP (ungrammatical) led to slower reading times than those obtained for matching antecedent-clitic pairs (grammatical). These differences arose at the clitic region in sentences with singular dependencies, and at the following region in sentences with plural dependencies.^4^ But in both cases, at the two-final regions ungrammatical sentences were read faster than grammatical ones. This is probably because, once participants detected the ungrammaticality of the sentence at previous regions (CW or CW+1), they simply speeded up reading the sentence to complete the grammaticality judgment task.

Grammaticality judgment accuracy data indicate that attraction effects occur both in grammatical and ungrammatical sentences in antecedent-clitic dependencies, but no significant effects were found in reading times. Reading time measures revealed no attraction effects, and we argue this is because they were obscured by the carry-over effects of the processing of the preceding plural attractor NPs. Hence, accuracy data suggests that clitic dependencies are affected by the same factors as subject-verb agreement, which would favor the *Clitics as Agreement Hypothesis* (e.g., Suñer, 1988; Franco, 2000) according to which clitics are agreement morphemes. In contrast, reading time data suggest that the processing of clitic dependencies is not affected by the same factors as subject-verb agreement and that might be processed differently (as suggested by Phillips et al., 2011; Dillon et al., 2013), which could be interpreted as evidence favoring the *Clitics as Pronouns Hypothesis*, according to which clitics are pronouns generated at the argument position that moved to the verb (e.g., Kayne, 1975; Torrego, 1988; Uriagereka, 1995; Sportiche, 1998; Anagnostopoulou, 2003; Marchis and Alexiadou, 2013, among others). In order to shed more light on this issue, in Experiment 2 we use ERP methods with which, due to their finer temporal resolution than self-paced reading methods, we might be able to detect the presence of (any) attraction effects that overcome the carry-over effects of the processing of plural attractor nouns.

## Experiment 2

Self-paced reading measures in Experiment 1 did not reveal attraction effects. Due to the finer temporal resolution of electrophysiological measures, in Experiment 2 we sought to detect attraction effects, if there are any, at clitic position. In this case, and following previous ERP evidence (Rossi et al., 2014), we expect clitic number violations to elicit a P600 component, which might also be preceded by a negative (N400 or LAN) component similar to the one reported for gender violations (Silva-Pereyra et al., 2012). Importantly, if clitics are agreement morphemes, we should be able to detect similar number attraction effects as those reported for subject-verb agreement (Kaan, 2002; Shen et al., 2013; Tanner et al., 2014, 2016), and we expect attraction effects to reduce the magnitude of the ERP components, particularly the P600.

### Method

#### Participants

Forty-six native speakers of Spanish (mean age 21.96 years; *SD* = 5.29), undergraduates at the University of the Basque Country (UPV/EHU), were paid for their participation. All participants were right-handed (Edinburgh Handedness Inventory, Oldfield, 1971) and they had normal or corrected to normal vision. All participants gave written informed consent under an experimental protocol approved by the Ethics Committee of the UPV/EHU (CEISH), in accordance with the Declaration of Helsinki.

#### Materials and Procedure

We used the same materials as in Experiment 1. However, in order to simplify the experimental design, only singular clitic dependencies were tested, and only four experimental conditions created, crossing two factors: Attractor Number (singular vs. plural) and Grammaticality (grammatical vs. ungrammatical object-clitics; see **Table 1**, examples 1–4). We created four lists containing 168 sentences, from which 48 were experimental sentences (12 per condition) and 120 were fillers (24 contained singular subject-verb agreement violations and 96 were grammatical sentences). Thus, only 28.6% of the sentences were ungrammatical (48 out of 168). All further details were the same as in Experiment 1.

The experiment was performed using Presentation^®^ software (Version 16.0^5^). Before the experiment started, participants were instructed about the EEG procedure and seated comfortably in a quiet room in front of a 17 inch monitor. All sentences were displayed in the middle of the screen word-by-word for 350 ms (ISI = 200 ms) in a rapid serial visual presentation paradigm. Materials were pseudo-randomized in the following way: no sentences of the same condition were displayed one after another and each experimental sentence was followed by a filler sentence. A fixation cross (+) indicated the beginning of each trial. After each sentence the words *CORRECTO* (‘correct’) and *INCORRECTO* (‘incorrect’) appeared on screen for 3000 ms, asking subjects to press one of two buttons (left or right, with response hand counterbalanced across participants) depending on whether the previously displayed sentence was grammatical or not. All 168 sentences were distributed over four blocks. Participants could take short breaks between blocks. Before the experiment began, participants ran a four trial procedure familiarization session. They were instructed not to blink or move when sentences were displayed and to make the grammaticality judgment as fast as possible. The whole session lasted no longer than 1 h.

#### EEG Recording

The ERPs were recorded from 32 scalp electrodes mounted in an elastic cap (Electro-Cap International, Inc.; 10–20 system). The electrodes were placed as follows: Fp1, Fpz, Fp2, F7, F3, Ground electrode, FZ, F4, F8, C5A, C1A, C2A, C6A, T3, C3, CZ, C4, T4, TCP1, C1P, C2P, TCP2, T5, P3, PZ, P4, T6, P1P, P2P, O1, Oz, and O2. All electrodes were referenced to left and right mastoids and rereferenced off-line to the nasal-bone electrode. The vertical and horizontal electro-oculograms (VEOG and HEOG) were recorded from electrodes located below (VEOG) and at the outer canthus (HEOG) of the right eye. Electrode impedance was kept below 10 kΩ. The electrical signals were digitalized on-line at a rate of 250 Hz and filtered off-line with a bandpass of 0.1–35 Hz (half-amplitude cut-offs). After the stimuli were recorded, the artifact rejection procedure was applied (off-line) in order to exclude periods containing eye blinks, head movements or technical artifacts from the data analysis.

#### Data Analysis

The same type of analysis as in Experiment 1 was performed for the behavioral data analysis, with the difference that models only included participants as a unique random effect (item random effect could not be added due to coding limitations in the ERP experimental design). The maximal random effect structure justified by model comparison included a by-participant Grammaticality random slope in the error and response latency analyses. For the electrophysiological data, ANOVA analyses were performed. Average ERPs were computed for each word and each electrode and the 200 ms pre-stimulus baseline was used. Trials with artifacts were excluded from averages. For statistical analyses 9 regions of interest (ROI) were generated, 6 for lateral and 3 for midline electrodes: left frontal (F7, F3, C5A), left central (T3, C3, TCP1), left parietal (T5, P3, O1), right frontal (F4, F8, C6A), right central (C4, T4, TCP2) and right parietal (P4, T6, O2). Midline electrodes were analyzed separately and three ROIs were created for them: frontal (C1A, FZ, C2A), central (C1P, Cz, C2P) and parietal (P1P, Pz, P2P).

As for lateral electrodes, an overall ANOVA was performed for the four within-subject variables included in the analyses: Attractor Number (singular vs. plural), Grammaticality (grammatical vs. ungrammatical), Hemisphere (left vs. right) and Region (frontal vs. central vs. posterior). Midline electrodes analysis included Region (central frontal vs. central vs. central posterior), Attractor Number (singular vs. plural), and Grammaticality (grammatical vs. ungrammatical), and they were analyzed separately from lateral electrodes. Further statistical analyses (MANOVAs) were conducted for each particular ROI whenever appropriate. Effects for Hemisphere or Region factors were only reported when they interacted with any of the main experimental manipulations: Attractor Number and Grammaticality.

Since ERPs are very sensitive to differences in the context preceding the critical region, our main analysis focused on the ERP components elicited by grammaticality effects in sentences containing singular and plural attractors, separately (examples 1 vs. 2; and 3 vs. 4 in **Table 1**, respectively). However, in order to explore asymmetric grammatical effects on attraction (Wagers et al., 2009; Shen et al., 2013), we also compared the main number attraction effects in grammatical and ungrammatical sentences separately (examples 1 vs. 3 and 2 vs. 4 in **Table 1**).

### Results

#### Grammaticality Judgment Errors

Analyses revealed significant Attractor Number (β = 0.626, *SE* = 0.074, *z* = 8.403, *p* < 0.001), with more errors produced in sentences containing number mismatching plural attractors than in sentences containing number matching singular attractors. The significant Attractor Number by Grammaticality interaction (β = 0.188, *SE* = 0.074, *z* = 2.528, *p* = 0.011) revealed that attraction effects were larger in ungrammatical sentences (β = 0.814, *SE* = 0.104, *z* = 7.803, *p* < 0.001) than in grammatical ones (β = 0.438, *SE* = 0.106, *z* = 4.111, *p* < 0.001; see **Table 5**).

**Table 5:** Raw count of errors (from a total of 548 responses per condition; percentages in brackets) and reaction time (ms) values of participants’ performance in the grammaticality judgment task in each experimental condition of Experiment 2.

	Grammaticality judgment errors		Response latencies	
		
	Grammatical	Ungrammatical	Grammatical	Ungrammatical
**Singular object**				
Singular attractor (match)	36 (5.8%)	37 (6.0%)	749	564
Plural attractor (mismatch)	77 (13.9%)	128 (27.4%)	775	699
*Attraction effect*	*-41 (8.1%)*	*-91 (21.4%)*	*-26*	*-134*


#### Grammaticality Judgment Response Latencies

The main effects of Attractor Number (β = 0.048, *SE* = 0.012, *t* = 3.975, *p* < 0.001) and Grammaticality were significant (β = -0.117, *SE* = 0.018, *t* = -6.499, *p* < 0.001), showing that participants were faster judging sentences containing number mismatching than matching attractors and were also faster rejecting ungrammatical sentences than accepting grammatical ones. The significant Attractor Number by Grammaticality interaction (β = 0.042, *SE* = 0.012, *t* = 3.458, *p* < 0.001) revealed attraction effects only when ungrammatical sentences had to be rejected (β = 0.095, *SE* = 0.017, *t* = 5.361, *p* < 0.001; with slower responses for sentences containing number mismatching than matching attractors.

#### ERP Results

Based on visual inspection and on previous ERP studies (Kaan, 2002; Chen et al., 2007; Severens et al., 2008; Dillon et al., 2013; Shen et al., 2013; Tanner et al., 2014, 2016), three main time windows were chose for statistical analyses at the *Clitic region*: 300–500 ms; 500–700 ms; and 700–900 ms.

##### 300–500 ms time window

**FIGURE 3:**
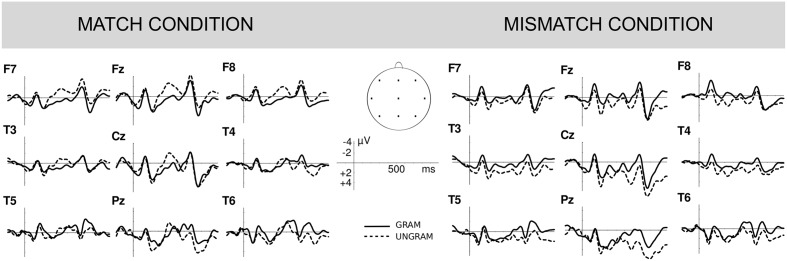
Grammaticality effects. Grand average event-related potentials time locked to the clitic (CW) position showing the main grammaticality effects for sentences containing number matching singular attractors **(left)** and number mismatching plural attractors (**right**; Experiment 2). The continuous lines represent grammatical sentences and the dotted lines represent ungrammatical sentences. Negativity is plotted upward and positivity is plotted downward.

At both the lateral and the midline electrodes, the Attractor Number by Grammaticality by Region three-way interactions were significant Lateral: *F*(2,90) = 5.59; *p* = 0.015; Midline: *F*(2,90) = 7.83; *p* = 0.004. To better understand this interaction we conducted follow-up analyses examining the mean Grammaticality effects in sentences containing number matching singular and number mismatching plural attractors separately at each ROI (see **Figure 3**). In sentences with number matching singular attractors, a larger negativity was found over frontal (but no central or posterior) sites of the scalp for ungrammatical sentences in both lateral electrodes [Frontal: *F*(1,45) = 10.44; *p* = 0.002; Central and Posterior: both *p*s > 0.1], and midline electrodes [Frontal: *F*(1,45) = 7.39, *p* = 0.009; Central and Posterior: both *p*s > 0.1] as compared to grammatical sentences. No statistically significant Grammaticality effect obtained in sentences with number mismatching plural attractors at any region, neither in lateral nor midline electrodes.

**FIGURE 4:**
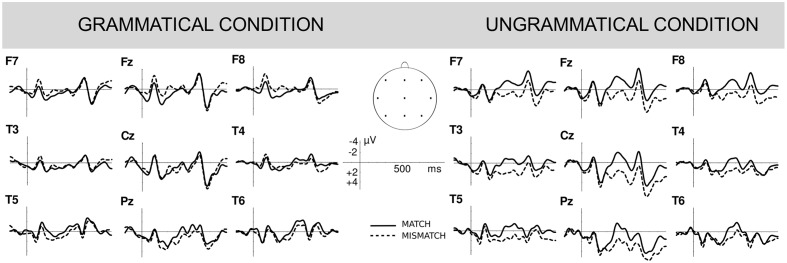
Attraction number effects. Grand average event-related potentials time locked to the clitic (CW) position showing the main attraction number effects for grammatical **(left)** and ungrammatical **(right)** sentences (**right**; Experiment 2). The continuous lines represent sentences containing number matching singular attractors and the dotted lines represent sentences containing number mismatching plural attractors. Negativity is plotted upward and positivity is plotted downward. Note that this figure just represents the same data plotted in **Figure 3** from a different view (focusing on main attraction effects instead of main grammaticality effects).

So far we focused on the main grammaticality effects elicited by sentences containing number matching and number mismatching attractors. However, in order to separately assess the presence of an asymmetrical grammaticality of attraction effect, we also conducted *complementary analyses* that focused on Attractor Number effects (see **Figure 4**). No statistically significant attraction effects obtained in grammatical sentences at any region, in either lateral or midline electrodes (all *p*-values > 0.05). However, significant attraction effects obtained in ungrammatical sentences, with larger negativity in number matching than in number mismatching conditions over all regions, both in lateral electrodes [Frontal: *F*(1,45) = 8.40, *p* = 0.006; Central: *F*(1,45) = 8.42, *p* = 0.006; Posterior: *F*(1,45) = 6.55; *p* = 0.014], and in midline electrodes [Frontal: *F*(1,45) = 9.11, *p* = 0.004; Central: *F*(1,45) = 10.16, *p* = 0.003; Posterior: *F*(1,45) = 6.81, *p* = 0.012].

##### 500–700 ms time window

At the lateral and midline electrodes, the two-way interaction of Grammaticality by Region was significant at 500–700 ms [Lateral: *F*(2,90) = 12.46, *p* < 0.001; Midline: *F*(2,90) = 7.26, *p* = 0.006] indicating a different electrophysiological response to grammatical vs. ungrammatical stimuli over frontal, central and posterior sites of the scalp. To better understand this interaction we conducted follow-up analyses examining the mean Grammaticality effects over the different regions of the scalp. This analysis showed a larger positivity for ungrammatical sentences than grammatical ones over central and posterior, but non-significant effects at frontal sites [Lateral electrodes: Frontal: *F*(1,45) = 0.10, *p* = 0.894; Central: *F*(1,45) = 4.04, *p* = 0.051; Posterior: *F*(1,45) = 6.66, *p* = 0.013; Midline electrodes: Frontal: *F*(1,45) = 1.19, *p* = 0.282; Central *F*(1,45) = 2.52, *p* = 0.120, Posterior: *F*(1,45) = 4.18, *p* = 0.047]. In addition, a main effect of Attractor Number was observed, with larger negativity over all electrode sites for sentences containing number matching singular attractors vs. number mismatching plural attractors [Lateral: *F*(1,45) = 5.30, *p* = 0.026; Midline: *F*(1,45) = 5.05, *p* = 0.030]. None of the interactions involving the Attractor Number factor yielded significance, suggesting that the distribution of the significant main grammaticality effects reported above were similar for sentences containing number matching singular attractors [Grammaticality × Region in Lateral: *F*(2,90) = 9.47, *p* = 0.002; and Midline electrodes: *F*(2,90) = 6.86, *p* = 0.007] and number mismatching plural attractors [Grammaticality × Region in Lateral electrodes: *F*(2,90) = 3.81, *p* = 0.049; and Grammaticality effect in Midline electrodes: *F*(1,45) = 6.52, *p* = 0.016].

For the sake of completeness, we also performed *complementary analyses* to separately examine the mean Attractor Number effects in grammatical and ungrammatical sentences. In grammatical sentences, none of the effects approached significance at any site (all *F*s < 1). In contrast, in ungrammatical sentences a main effect of Attractor Number was found over the lateral and midline sites [Lateral *F*(1,45) = 5.43, *p* = 0.024; midline: *F*(1,45) = 5.56, *p* = 0.023], revealing that the larger negativity elicited by number matching singular attractors vs. number mismatching plural ones at the 300–500 ms time window continued to be significant at the 500–700 ms time window. This effect was no longer significant at the 700–900 ms time window (Lateral and Midline: both *p*s > 0.1).

##### 700–900 ms time window

At lateral and midline electrodes, the same Grammaticality by Region interaction pattern reported for the 500–700 ms time window was found [Lateral: *F*(2,90) = 16.38, *p* < 0.001; Midline: *F*(2,90) = 11.74, *p* = 0.001]. The analyses of Grammaticality effects replicated the pattern reported in the 500–700 ms time window: [Lateral electrodes: Frontal: *F*(1,45) = 0.27, *p* = 0.609; Central: *F*(1,45) = 3.43, *p* = 0.07; Posterior: *F*(1,45) = 7.42, *p* = 0.009; Midline electrodes: Frontal: *F*(1,45) = 0.39, *p* = 0.536; Central *F*(1,45) = 1.81, *p* = 0.185; Posterior: *F*(1,45) = 5.01, *p* = 0.030]. However, in the 700–900 ms time window the main effect of Attractor Number was not significant [Lateral: *F*(1,45) = 1.43, *p* = 0.237; Midline: *F*(1,45) = 1.79, *p* = 0.188]. None of the interactions involving Attractor Number factor yielded significance, indicating similar distribution of the grammaticality effects reported above in sentences containing number matching singular [Grammaticality × Region in Lateral: *F*(2,90) = 11.31, *p* = 0.001; and Midline electrodes: *F*(2,90) = 8.20, *p* = 0.003] and number mismatching plural attractors [Grammaticality × Region in Lateral: *F*(2,90) = 5.343, *p* = 0.020; and Midline electrodes: *F*(2,90) = 4.08, *p* = 0.039].

Finally, none of the *complementary analyses* focused on examining the mean Attractor Number effects in grammatical and ungrammatical sentences yielded significance at any site (all *p*s > 0.1).

## General Discussion

In two experiments, we investigated the effects of number attraction on Spanish object clitic dependencies, elicited by number mismatching attractor NPs intervening between the clitic and its antecedent. In Experiment 1, grammaticality judgment accuracy data revealed number attraction effects and number markedness effects, since attraction effects were detected only when the antecedent-clitic dependency was singular, replicating the number markedness effect reported in agreement dependencies (Bock and Miller, 1991; Eberhard, 1997; Pearlmutter et al., 1999; Pearlmutter, 2000; Wagers et al., 2009). However, on-line reading times failed to reveal attraction effects, possibly because of the greater carry-over effect of the slow down originated while reading the plural attractor NPs. In Experiment 2, number attraction effects were detected both by grammaticality judgment data and electrophysiological measures. Grammaticality judgment accuracy and response time data revealed number attraction effects in antecedent-clitic dependency resolution, since there were more errors and slower RTs in sentences containing number mismatching attractors vs. number matching ones. Additionally, asymmetrical attraction was observed, that is, attraction effects where larger for ungrammatical sentences than for grammatical ones (replicating Franck et al., 2015: Experiment 3). As discussed next, these patterns of results were also replicated by the ERP data.

### Electrophysiological Indexes of Antecedent-Clitic Dependencies and Number Attraction

In Experiment 2, violations in sentences with singular attractors (e.g., *…el paquete para el vecino ^∗^los…*) elicited a frontal negativity followed by a P600 component. These components have been previously reported for antecedent-clitic dependency violations, but not simultaneously: Silva-Pereyra et al. (2012) report an N400 for feminine gender violation and a P600 for masculine gender violation, while Rossi et al. (2014) report a P600 for both gender and number violations. Our biphasic ERP pattern replicates the one usually reported for agreement violations (see Molinaro et al., 2011) and other types of pronominal dependency violations such as reflexives or subject pronouns (Schmitt et al., 2002; Hammer et al., 2005, 2008; Lamers et al., 2006).

Regarding number attraction effects, violations involving singular antecedents and plural clitics with intervening plural attractors elicited a P600 component with no trace of a preceding negativity (e.g., *el paquete para los vecinos ^∗^los…*). This pattern of results, together with those from grammaticality judgment accuracy data in Experiments 1 and 2, reveals greater difficulty detecting clitic number violations when a mismatching plural attractor intervenes. We interpret the absence of a negative component as signaling an attraction effect due to the mismatching attractor (replicating Chen et al., 2007; Severens et al., 2008; Shen et al., 2013). We did not find a reduction of the amplitude of the P600 component that could be interpreted as evidence for attraction effects, as in some studies on agreement (Kaan, 2002; Shen et al., 2013; Tanner et al., 2014).

Importantly, our results also revealed electrophysiological indexes of *asymmetrical* attraction effects: attraction effects only occurred in ungrammatical sentences, not in grammatical ones. In ungrammatical sentences, plural clitics with singular antecedents elicited a large and broadly distributed negativity when preceded by plural attractors, as compared to those preceded by number matching singular attractors. No equivalent differences were found for grammatical sentences. These results converge with grammaticality judgment accuracy and response time data in Experiment 2, where weaker number attraction effects obtained for grammatical sentences as compared to ungrammatical ones. Importantly, these asymmetrical effects suggest that they are in fact due to attraction and not to carry-over effects originated while reading the preceding plural attractors, as might have occurred in the self-paced reading task. If the effects shown in ungrammatical sentences were due to carry-over effects, they should also have been detected in grammatical ones.

In sum, behavioral and ERP results from Experiment 2 showed that antecedent-clitic dependencies are also subject to attraction effects and that these effects are detected in ungrammatical sentences only. Our ERP results identified frontal negative components as the main electrophysiological indexes of attraction effects.

### On Self-paced Reading vs. ERP Data

The fact that no clear attraction effects obtained in reading times for antecedent-clitic dependencies (either at clitic position or following word regions) suggests that this type of dependencies are resilient to attraction effects, as previously revealed by Dillon et al. (2013) and Parker and Phillips (2017). However, in Experiment 2 these effects were detectable by methods with finer temporal resolution such as ERPs. Off-line grammaticality judgment measures showed attraction effects in both experiments, but asymmetrical attraction effects were only obtained in Experiment 2. Certain experimental design variables might have contributed to these differences. For instance, in Experiment 1 grammatical and ungrammatical sentences containing plural antecedents were included, so that a grammaticality judgment task could be performed with sentences containing plural clitics. In contrast, in Experiment 2 all sentences with plural clitics were ungrammatical. Although some researchers counterbalanced this by adding as fillers grammatical sentences containing plural controllers (e.g., Franck et al., 2015: in all three Experiments), many self-paced reading and ERP studies do not (Pearlmutter et al., 1999: Experiments 1 and 2; Shen et al., 2013; Tanner et al., 2014: in all three Experiments), or do not report it (Chen et al., 2007; Lago et al., 2015: in all four Experiments; Wagers et al., 2009: Experiments 2, 4, 5, and 6). The fact that in Experiment 2 a plural clitic was a perfectly reliable signal of an ungrammatical sentence might have lessened the capacity of attractors to elicit attraction. Although the confound between grammaticality and the processing of plural clitics might have had some effect, we believe this cannot be the determinant factor behind the results of Experiment 2. This is because we should expect similar electrophysiological response patterns related to grammaticality (or clitic number) effects in sentences containing number matching and mismatching attractors. However, we report different ERP patterns in grammatical vs. ungrammatical sentences: frontal negativity-P600 vs. only P600, respectively. If participants used a plural-clitic equal ungrammatical task-specific strategy, attraction effects might have diminished overall, augmenting the possibility to detect asymmetrical attraction effects. The impact these design differences might have had on the off-line results obtained in both experiments seem to be reflected in our on-line measures too, where ERP data provided finer grained timing effects than the self-paced reading data. Next, we discuss the main implications of these findings for current models of language processing.

### Fitting the Findings with Models of Agreement Processing

The negative components (e.g., LAN/N400) reported above have been generally interpreted as indexing a greater difficulty to integrate the predicted critical word into the previous context (Friederici et al., 1993; Münte et al., 1993; Friederici, 2002; Rossi et al., 2005). In the case of antecedent-clitic dependencies (e.g., *…el paquete para el/los vecino(s) ^∗^los…*), attraction effects modulate these negative components, revealing a greater difficulty to predict/integrate a plural clitic (^∗^*los*) that disagrees in number with the singular antecedent (*el paquete*) when it was preceded by a singular attractor (*el vecino*), as compared to when it was preceded by a plural attractor (*los vecinos*).

These results can be accounted for under the cue-based retrieval and similarity-based interference accounts of dependency processing (Lewis and Vasishth, 2005; Wagers et al., 2009). In these models, when the number feature of the dependent element (in our case, the clitic) is encountered, a retrieval mechanism searches for a matching element stored in working memory (in this case, the antecedent NP). Accordingly, the grammaticality effect reported here ensues: when a plural clitic is encountered in a context where all possible antecedents are singular, the predictability and/or integration of the clitic is most difficult, eliciting a frontal negativity. But when a plural clitic is encountered after a plural attractor, the similarity-based interference of the plural attractor with the singular antecedent to be retrieved from memory leads to erroneously interpreting the plural attractor as the antecedent of the clitic, so that no frontal negativity is elicited.

Conversely, the asymmetric effect emerges because, in grammatical sentences, the number mismatching attractor is not retrieved from memory, either because the retrieval mechanism is not deployed or because it only retrieves antecedent candidates that fully match the features of the clitic. In other words, when the number of the antecedent and the dependent clitic match (i.e., in grammatical sentences), it is assumed that the number of the attractor noun is not retrieved, so that no ERP differences ensue for matching and mismatching attractors. In contrast, when the number of the antecedent and the clitic do not match (i.e., in ungrammatical sentences), a reanalysis process ensues. Thus, a larger frontal negativity is expected in sentences containing number matching attractors as compared to mismatching ones, because illusions of grammaticality only occur in sentences where plural attractor NPs match the number of the plural clitic and can be mistakenly retrieved as their antecedents (Wagers et al., 2009; Phillips et al., 2011). Our results fully support these predictions.

The absence of a frontal negativity in sentences containing mismatching plural attractors is an index of the presence of number attraction effects during antecedent-clitic dependency resolution. Additionally, the asymmetric effect revealed by the absence of ERP components indexing attraction in grammatical sentences (in contrast to the negative component elicited in ungrammatical ones), provides compelling evidence in support of cue-based retrieval models as accounts of attraction effects in comprehension (Wagers et al., 2009; Shen et al., 2013). Unfortunately, our data cannot adjudicate between the possibility that encountering a singular clitic out-competes retrieval of plural antecedent candidates so that the attractor is not retrieved, and the possibility that the dependency is correctly processed without the deployment of retrieval mechanisms. Importantly, the present results cannot be accommodated into feature percolation models because they assume attraction effects are driven by an erroneous number representation in the antecedent NP. Hence, they predict equivalent effects in grammatical and ungrammatical sentences (Nicol et al., 1997; Pearlmutter et al., 1999), contrary to our findings (see also, Wagers et al., 2009; Dillon et al., 2013; Lago et al., 2015). Electrophysiological evidence in Experiment 2 revealed a clear asymmetrical effect of attraction. In sum, the evidence here provides strong support for cue-based retrieval models of dependency resolution in language processing, and are incompatible with alternative feature percolation accounts.

Finally, in addition to the absence of frontal negative components as an electrophysiological index of attraction effects, we also reported that number violations elicited a P600 component both when sentences contained a matching singular attractor and when they contained a mismatching plural attractor. This in turn reveals that, despite the presence of attraction effects, participants could detect the ungrammaticality of sentences containing number violations. These findings contrast with those reported in Shen et al. (2013), where attraction effects led to the absence of associated ERP components, and in Kaan (2002) and Tanner et al. (2014), where attraction effects caused a reduction in the amplitude of the ungrammaticality/reanalysis related P600. In the former case, the differences in results likely originate from task differences: the grammaticality judgment task we used required participants to explicitly and consciously check and reanalyze sentences for well-formedness, which would encourage the appearance of the P600 even in sentences where number attraction effects occurred, while the comprehension task used by Shen et al. (2013) did not require participants to pay attention to grammaticality. However, the differences between our results and those of Kaan (2002) and Tanner et al. (2014), where attraction effects reduced the amplitude of the P600 component, are harder to explain based on task differences, given that all studies used a grammaticality judgment task. The main differences with regard to our study involves a smaller ratio of ungrammatical sentences (28.6% in our study vs. 50% in theirs); the type of phenomenon explored, where we studied attraction effects in antecedent-clitic dependencies and they did so in subject-verb agreement. These findings might tentatively be interpreted as evidence that antecedent-clitic dependencies tap into processes different from those involved in agreement.

### What Do These Findings Reveal about the Nature of Clitic Dependencies?

Whether Romance clitics are pronouns or agreement morphemes is under debate. Although our experimental approach does not provide direct evidence supporting either type of syntactic analysis, we believe it offers indirect evidence that can be informative. Some studies compared the magnitude of attraction effects elicited in different types of dependencies (Dillon et al., 2013; Parker and Phillips, 2017) and observed that antecedent-reflexive pronoun dependencies were more resilient to attraction than subject-verb agreement. We suggest that these differences correlate with the distinct nature of these two types of dependencies. Although both types of dependencies rely on similar cue-based retrieval mechanisms, antecedent-pronoun dependencies involve a referential dependency between two nominal arguments and weight structural cues more strongly than morphological ones, precluding the erroneous retrieval of non-licensed antecedent candidates. In contrast, subject-verb agreement is a morphological mechanism used to index the arguments of sentences where morphological cues weigh more than structural ones, making the erroneous retrieval of non-licensed attractors possible.

Although a direct comparison of attraction effects between subject-verb and antecedent-clitic dependencies goes beyond the scope of this study, we argue that our results align better with results previously obtained for antecedent-reflexive pronoun than for subject-verb agreement dependencies. Subject-verb agreement resolution shows consistent attraction effects in self-paced reading studies and these effects have been shown to mainly modulate late positive P600 components in ERP experiments. In contrast, antecedent-clitic dependency resolution is resilient to attraction effects in self-paced reading (Experiment 1; replicating reflexive pronoun studies: Dillon et al., 2013; Parker and Phillips, 2017) and affected early frontal negative ERP components (Experiment 2). Hence, we tentatively interpret the observed resilience of antecedent-clitic dependencies to attraction effects and the fact that they modulate different electrophysiological components than in subject-verb agreement to indicate that antecedent-clitic and verb agreement dependencies constitute different types of linguistic dependencies. Thus, we interpret our indirect evidence to favor the *Clitics as Pronouns Hypothesis* originally proposed by Kayne (1975), which suggests that Spanish object-clitics are processed as pronominal elements.

Regarding the ERP patterns indexing attraction effects in antecedent-clitic dependencies, we observed that attraction effects lead to the absence of a frontal negativity, while in previous studies subject-verb agreement attraction effects modulated the later positive P600 component (Kaan, 2002; Tanner et al., 2014). Although we reckon that linking the modulation of different ERP components as evidence for different types of processes might be seen as speculative, we tentatively argue the difference in the type of associated components signals the different processes involved in the resolution of pronominal and agreement dependencies: attraction effects revealed by frontal negativities might be related to pronominal processing and active retrieval of the lexical representation of possible antecedents and signals difficulty of syntactic/semantic integration of the full arguments (i.e., difficulty of establishing antecedent-clitic pronominal dependencies) (Barkley et al., 2015). Thus, in ungrammatical sentences, no negative component appeared because the plural attractor NP might have been incorrectly identified as the plural clitic antecedent NP. In contrast, attraction effects revealed at later positivities might be mainly related to purely morphological processes like agreement and signal difficulty to integrate and reanalyze morphological features (e.g., in verb agreement dependencies) (Hagoort et al., 1993).

Certainly, further research making direct comparisons between antecedent-clitic and subject-verb agreement dependencies in relatively similar syntactic contexts (i.e., distance between the two agreeing elements, structural position of the elements, whether they are in their canonical position or not, etc.) will help to better identify the processes underlying both structures. Further research ought to provide a fuller and more systematic picture of the main electrophysiological indexes involved in the resolution of the different types on linguistic dependencies across a wider array of languages.

## Conclusion

We provide novel evidence regarding the electrophysiological indexes associated to processing mechanisms underlying attraction effects in the comprehension of antecedent-clitic dependencies. Our results show that antecedent-clitic dependencies can be disrupted by an intervening attractor. Studying this pattern of disruption, we replicate the grammatical asymmetry of attraction effects observed in subject-verb agreement (Wagers et al., 2009; Shen et al., 2013; Tanner et al., 2014, 2016), which supports cue-based retrieval mechanisms of attraction. Finally, despite being resilient to attraction effects in self-paced reading measures, clitic dependencies show electrophysiological indexes of attraction that involve components different from those commonly found for verb agreement (frontal negativities for clitics and late positivities for agreement). These differences, we speculate, suggest that clitic-pronoun and verb agreement dependencies involve distinct processing routines for their resolution. Further research involving more languages and types of dependencies will undoubtedly contribute to shed more detail in this general picture of dependency-processing in language comprehension.

## Author Contributions

MS and AZ performed research and analyzed data. MS, AZ, KE, and IL designed, discussed and interpreted findings and wrote the paper.

## Conflict of Interest Statement

The authors declare that the research was conducted in the absence of any commercial or financial relationships that could be construed as a potential conflict of interest.

## Footnotes

1 Note that in these types of structures the object NP (*el paquete para los vecinos*, in the example of **Table 1**) might have been interpreted as a subject which might have predicted the following word to be a verb (*el paquete para los vecinos era…*, “the package for the neighbors was*…*”) instead of a left-dislocated object clitic (*el paquete para los vecinos lo …, lit.* “the package for the neighbors it *…*”). Due to this ambiguity, one might argue that the NP would only be recognized as an object NP when the verb is reached, and thus participants might be stuck in a garden-path until then. However, since in our materials all the NPs were inanimate, they would have been most likely interpreted as the subjects of an intransitive event, because the subjects of transitive events are more likely to be animate. Thus, reading at the critical region an object clitic instead of a verb would suffice to break the garden-path effect, as it would strongly prioritize interpreting the antecedent NP as an object NP. Importantly, it is worth noting that in the event the antecedent NP was interpreted as a subject NP, the noun inside the PP (*el/los vecino/s*) could not be considered the antecedent of the object clitic (*lo/s*) in Peninsular Spanish. We thank Brian Dillon for pointing out to this possible confound.

2 The spill-over effects were computed following the steps described in Florian Jaeger’s blog: http://www.hlplab.wordpress.com/2008/01/23/modeling-self-paced-reading-data-effects-of-word-length-word-position-spill-over-etc/ and https://hlplab.wordpress.com/2007/11/23/spill-over-effects-in-self-paced-reading/; accessed June 7, 2017).

3 Note that the models with or without the by-item Attractor Number random slope revealed the same patterns of results in all regions (even if the models showed overparameterization in regions R5, R6, R7, CW+2, and CW+3). Analysis with log-transformed residual reading times as a dependent variable also showed similar results as the ones reported with log-transformed reading times.

4 The fact that grammaticality effects were already detected at the critical region in singular object antecedent conditions suggests that the possible garden path effects elicited while reading the ambiguous antecedent NP were solved at the critical object clitic region, without the need to wait until the following verb region (see Footnote 1).

5 www.neurobs.com
