# Supplementary material for: On the Nature of Clitics and Their Sensitivity to Number Attraction Effects
Source: Front Psychol. 2017 Sep 5;8:1470. doi: 10.3389/fpsyg.2017.01470 (PMC5591828; doi:10.3389/fpsyg.2017.01470)
Supplement: Supplementary file 1 [file Data_Sheet_1.pdf]

Appendix

Materials used in Experiments 1 and 2. In the examples, singular object nouns, attractor nouns and object clitics are presented before the slash and plural object nouns, attractor nouns and object clitics are presented after the slash. Thus, the eight versions of each trial can be created in the following way (in Experiment 2, only conditions A, B, C, and D were used): (A) *El cartero afirmó que el paquete para el vecino lo entregó a tiempo* (Singular Object, Singular Attractor, Grammatical); (B) *El cartero afirmó que el paquete para el vecino *los entregó a tiempo* (Singular Object, Singular Attractor, Ungrammatical); (C) *El cartero afirmó que el paquete para los vecinos lo entregó a tiempo* (Singular Object, Plural Attractor, Grammatical) (D) *El cartero afirmó que el paquete para los vecinos *los entregó a tiempo* (Singular Object, Plural Attractor, Ungrammatical); (E) *El cartero afirmó que los paquetes para los vecinos los entregó a tiempo* (Plural Object, Plural Attractor, Grammatical); (F) *El cartero afirmó que los paquetes para los vecinos *lo entregó a tiempo* (Plural Object, Plural Attractor, Ungrammatical); (G) *El cartero afirmó que los paquetes para el vecino los entregó a tiempo* (Plural Object, Singular Attractor, Grammatical); (H) *El cartero afirmó que los paquetes para el vecino *lo entregó a tiempo* (Plural Object, Singular Attractor, Ungrammatical).

1. El cartero afirmó que el paquete/los paquetes para el vecino/los vecinos (*)lo/(*)los entregó a tiempo.
   The postman affirmed that the package/s for the neighbor/s (he) delivered (*)it/(*)them on time.

2. El albañil contestó que el trabajo/los trabajos para el director/los directores (*)lo/(*)los aceptó por dinero.

   The construction worker answered that the work/s for the director/s (he) accepted (*)it/(*)them for money.

3. El agricultor opina que el camino/los caminos hacia el monte/los montes (*)lo/(*)los conoce muy bien.

   The farmer believes that the path/s towards the mountain/s (he) knows (*)it/(*)them very well.

4. El médico admite que el tratamiento/los tratamientos contra el dolor/los dolores (*)lo/(*)los inventó hace años.

   The doctor admits that the treatment/s against the pain/s (he) invented (*)it/(*)them years ago.

5. El piloto confirma que el aterrizaje/los aterrizajes en el desierto/los desiertos (*)lo/(*)los ejecutó con destreza.

   The pilot confirms that the landing/s in the desert/s (he) executed (*)it/(*)them with dexterity.

6. El sastre manifestó que el traje/los trajes para el bautizo/los bautizos (*)lo/(*)los hizo con prisa.

   The tailor manifested that the costume/s for the christening/s (he) made (*)it/(*)them in a hurry.

7. El taxista piensa que el viaje/los viajes con el jefe/los jefes (*)lo/(*)los planeó con tiempo.

   The taxi driver thinks that the travel/s with the boss/es (he) planned (*)it/(*)them ahead of time.

8. El veterinario admite que el medicamento/los medicamentos para el caballo/los caballos (*)lo/(*)los compró por necesidad.

   The veterinarian admits that the medicine/s for the horse/s (he) bought (*)it/(*)them out of necessity.

9. El bombero sostiene que el fuego/los fuegos en el bosque/los bosques (*)lo/(*)los apagó con dificultad.

   The firefighter maintains that the fire/s in the wood/s (he) extinguished (*)it/(*)them with difficulty.

10. El marinero opina que el aviso/los avisos sobre el accidente/los accidentes (*)lo/(*)los mandó muy tarde.

    The sailor believes that the warning/s about the accident/s (he) send (*)it/(*)them too late.

11. El panadero anunció que el pastel/los pasteles con el dibujo/los dibujos (*)lo/(*)los vendió muy rápido.

    The baker announced that the cake/s with the picture/s (he) sold (*)it/(*)them very fast.

12. El jugador confesó que el disparo/los disparos hacia el objetivo/los objetivos (*)lo/(*)los realizó con rapidez.

    The player confessed that the shoot/s to the target/s (he) made (*)it/(*)them quickly.

13. El explorador dice que el río/los ríos bajo el acantilado/los acantilados (*)lo/(*)los descubrió por casualidad.

    The explorer says that the river/s beneath the cliff/s (he) discover (*)it/(*)them casually.

14. El cazador dijo que el cepo/los cepos para el zorro/los zorros (*)lo/(*)los colocó de noche.

    The hunter says that the trap/s for the fox/es (he) placed (*)it/(*)them during the night.

15. El concejal remarcó que el informe/los informes contra el alcalde/los alcaldes (*)lo/(*)los presentó hace meses.

    The councilman remarked that the report/s against the mayor/s (he) presented (*)it/(*)them months ago.

16. El escritor cree que el libro/los libros sobre el actor/los actores (*)lo/(*)los terminará muy pronto.

    The writer thinks that the book/s about the actor/s (he) will finish (*)it/(*)them very soon.

17. El periodista subrayó que el artículo/los artículos contra el empresario/los empresarios (*)lo/(*)los publicó su director.

    The journalist emphasized that the article/s against the businessman/s (where) published (*)it/(*)them by his director.

18. El productor aclaró que el programa/los programas sobre el tenista/los tenistas (*)lo/(*)los presentarán pasado mañana.

    The producer clarified that the program/s about the tennis player/s (*)it/(*)them will be presented the day after tomorrow.

19. El ladrón declaró que el coche/los coches bajo el árbol/los árboles (*)lo/(*)los robó el viernes.

    The burglar declared that the car/s under the tree/s (he) stole (*)it/(*)them on Friday.

20. El empleado aclaró que el muro/los muros con el graffiti/los graffitis (*)lo/(*)los limpió en junio.

    The employee clarified that the wall/s with the graffiti/es (he) cleaned (*)it/(*)them on June.

21. El arquitecto contó que el edificio/los edificios con el espejo/los espejos (*)lo/(*)los diseñó en Londres.

The architect related that the building/s with the glass/es (he) designed (*)it/(*)them in London.

22. El abuelo dice que el paseo/los paseos por el lago/los lagos (*)lo/(*)los hace cada día.

The grandfather says that the walk/s in the lake/s (he) makes (*)it/(*)them every day.

23. El padre aclaró que el regalo/los regalos para el bebé/los bebés (*)lo/(*)los compró en rebajas.

The father clarified that the present/s for the baby/ies (he) bought (*)it/(*)them in sales.

24. El ministro explicó que el aviso/los avisos sobre el atentado/los atentados (*)lo/(*)los recibió a tiempo.

The minister explained that the warning/s about the attack/s (he) received (*)it/(*)them on time.

25. La pastora dijo que la casa/las casas de la montaña/las montañas (*)la/(*)las visitó en invierno.

The shepherdess said that the house/s in the mountain/s (she) visited (*)it/(*)them in Winter.

26. La chica contó que la historia/las historias sobre la aldea/las aldeas (*)la/(*)las escuchó muchas veces.

The girl related that the story about the small village (she) heard (*)it/(*)them many times.

27. La profesora dice que la crítica/las críticas sobre la novela/las novelas (*)la/(*)las escribió en vacaciones.

The teacher says that the review/s about the novel/s (she) wrote (*)it/(*)them on holidays.

28. La niñera mencionó que la enfermedad/las enfermedades de la perra/las perras (*)la/(*)las descubrió por casualidad.

The babysitter mentioned that the sickness/es of the female dog (she) discovered (*)it/(*)them by accident.

29. La actriz aclaró que la mansión/las mansiones bajo la colina/las colinas (*)la/(*)las compró hace poco.

The actress clarified that the mansion/s beneath the hill/s (she) bought (*)it/(*)them not long ago.

30. La abogada piensa que la demanda/las demandas contra la propietaria/las propietarias (*)la/(*)las rechazaron hace tiempo.

The lawyer believes that the lawsuit/s against the landlady/ies (they) rejected (*)it/(*)them long time ago.

31. La secretaria dice que la oficina/las oficinas de la empresa/las empresas (*)la/(*)las limpia a menudo.

The secretary says that the office/s of the enterprise/s (she) cleans (*)it/(*)them frequently.

32. La jueza aclaró que la multa/las multas a la encargada/las encargadas (*)la/(*)las aceptó a trámite.

The judge clarified that the fine/s to the manager/s (she) admitted (*)it/(*)them to procedure.

33. La cantante declaró que la canción/las canciones para la mujer/las mujeres (*)la/(*)las escribió por encargo.

The singer declared that the song/s for the woman/en (she) wrote (*)it/(*)them on request.

34. La directora dice que la queja/las quejas de la estudiante/las estudiantes (*)la/(*)las recibió por carta.

The director says that the complain/s of the student/s (she) received (*)it/(*)them by letter.

35. La zoóloga aclaró que la trampa/las trampas para la serpiente/las serpientes (*)la/(*)las colocó con cuidado.

The zoologist clarified that the trap/s for the snake/s (she) placed (*)it/(*)them carefully.

36. La mujer dice que la lámpara/las lámparas sobre la mesa/las mesas (*)la/(*)las limpió dos veces.

The woman says that the lamp/s on the table/s (she) cleaned (*)it/(*)them twice.

37. La señora contestó que la crema/las cremas para la verruga/las verrugas (*)la/(*)las utilizaba cada día.

The lady replied that the cream/s for the wart/s (she) used (*)it/(*)them every day.

38. La azafata manifestó que la opinión/las opiniones sobre la pasajera/las pasajeras (*)la/(*)las expresó sin recapacitar.

The stewardess declared that the opinion/s about the passenger/s (she) expressed (*)it/(*)them without thinking twice.

39. La cocinera afirma que la cena/las cenas para la familia/las familias (*)la/(*)las preparó con cariño.

The cook affirms that the dinner/s for the family/ies (she) made (*)it/(*)them with love.

40. La camarera sostiene que la cerveza/las cervezas para la chica/las chicas (*)la/(*)las sirvió como siempre.

The waitress holds that the beer/s for the girl/s (she) served (*)it/(*)them as usual.

41. La enfermera anunció que la cura/las curas contra la enfermedad/las enfermedades (*)la/(*)las aplicó con cuidado.

The nurse announced that the cure/s against the sickness/es (she) applied (*)it/(*)them with care.

42. La dentista admite que la máquina/las máquinas de la oficina/las oficinas (*)la/(*)las reparó dos veces.

The dentist admits that the machine/s of the office/s (she) repaired (*)it/(*)them twice.

43. La criada admite que la llave/las llaves de la despensa/las despensas (*)la/(*)las robó con premeditación.

The maid admits that the key/s of the larder (she) stole (*)it/(*)them with intent.

44. La traductora afirma que la novela/las novelas sobre la catedral/las catedrales (*)la/(*)las tradujo del inglés.

The translator affirms that the novel/s about the cathedral/s (she) translated (*)it/(*)them from English.

45. La psicóloga admite que la cena/las cenas para la niña/las niñas (*)la/(*)las encargó el lunes.

The psychologist admits that the dinner/s for the girl/s (she) ordered (*)it/(*)them on Monday.

46. La estudiante señaló que la clase/las clases sobre la revolución/las revoluciones (*)la/(*)las escuchó con atención.

The student pointed out that the class/es about the revolution/s (she) listened (*)it/(*)them with attention.

47. La pintora anunció que la sugerencia/las sugerencias sobre la obra/las obras (*)la/(*)las agradeció con sinceridad.

The painter announced that the suggestion/s about the work/s (she) thanked (*)it/(*)them honestly.

48. La psiquiatra admite que la descripción/las descripciones de la enfermedad/las enfermedades (*)la/(*)las envió muy tarde.

The psychiatrist admits that the description/s of the sickness/es (she) send (*)it/(*)them very late.
